# Suppression of Urokinase-Type Plasminogen Activator Receptor by Docosahexaenoic Acid Mediated by Heme Oxygenase-1 in 12-*O*-Tetradecanoylphorbol-13-Acetate-Induced Human Endothelial Cells

**DOI:** 10.3389/fphar.2020.577302

**Published:** 2020-11-26

**Authors:** Sen Lian, Shinan Li, Dhiraj Kumar Sah, Nam Ho Kim, Vinoth‐Kumar Lakshmanan, Young Do Jung

**Affiliations:** ^1^ Department of Biochemistry and Molecular Biology, School of Basic Medical Sciences, Southern Medical University, Guangdong, China; ^2^ Research Institute of Medical Sciences, Chonnam National University Medical School, Gwangju, Korea; ^3^ Centre for Preclinical and Translational Medical Research (CPTMR), Central Research Facility (CRF), Faculty of Clinical Research, Sri Ramachandra Institute of Higher Education and Research, Chennai, India; ^4^ Thumbay Research Institute for Precision Medicine and Department of Biomedical Sciences, Gulf Medical University, Ajman, United Arab Emirates

**Keywords:** docosahexaenoic acid (22:6ω-3), urokinase plasminogen activator receptor, heme oxygenase-1, carbon monoxide, cell invasion

## Abstract

Urokinase-type plasminogen activator receptor (uPAR) plays a crucial role in inflammation and tumor metastasis. Docosahexaenoic acid (DHA), a representative omega-3 polyunsaturated fatty acid, has been shown to exhibit anti-inflammatory and anti-tumor properties. However, the mechanism by which DHA negatively regulates uPAR expression is not yet understood. The aim of this study was to investigate the effect of DHA on 12-*O*-tetradecanoylphorbol-13-acetate (TPA)-induced uPAR expression and potential role of heme oxygenase-1 (HO-1) in DHA-induced inhibition of uPAR in human endothelial ECV304 cells. Results showed that TPA induced uPAR expression in a time dependent manner, while DHA inhibited uPAR expression in a concentration-dependent manner. Moreover, treatment with DHA induced HO-1 expression in a time- and concentration-dependent manner. In addition, DHA-induced inhibition of uPAR expression and cell invasion in TPA-stimulated cells was reversed by si-HO-1 RNA. Induction of HO-1 by ferric protoporphyrin IX (FePP) inhibited TPA-induced uPAR expression, and this effect was abolished by treatment with the HO-1 inhibitor tin protoporphyrin IX (SnPP). Additionally, carbon monoxide, an HO-1 product, attenuated TPA-induced uPAR expression and cell invasion. Collectively, these data suggest a novel role of DHA-induced HO-1 in reducing uPAR expression and cell invasion in human endothelial ECV304 cells.

## Introduction

Cancer is among the leading causes of death in both economically developed and developing countries (Mathers et al., 2008). Tumor metastasis is the leading cause of cancer-related mortality. Urokinase-type plasminogen activator (uPA), its inhibitors, and Urokinase-type plasminogen activator receptor (uPAR) form a complex proteolytic system, which has been related to tumor metastasis (Mazar et al., 1999). uPAR has been shown to be involved in nearly every step of cancer metastasis, including cell migration (Schiller et al., 2009), adhesion (Andreasen et al., 1997; Liang et al., 2008), angiogenesis (Mignatti and Rifkin, 1995), and invasion (Subramanian et al., 2006; Kunigal et al., 2007). Hence, uPAR is thought to be an important regulator of the invasive properties of cancer cells (Blasi et al., 1987). Therefore, agents with the ability to block uPAR expression may serve as potential candidates for the treatment of human cancers.

Docosahexaenoic acid (DHA) is a major ω-3 polyunsaturated fatty acids enriched in fatty fish and fish oil supplements possesses anti-inflammatory and anti-cancer properties (Simopoulos, 2002; Whyte et al., 2012). With respect to the anti-cancer effect, DHA treatment was reported to inhibit MMP-9 expression in human breast cancer cells, MCF-7 (Chen et al., 2013). Treatment with DHA has also been shown to inhibit vascular sprout formation in retinal microvascular endothelial cells (Matesanz et al., 2010). Moreover, epidemiological evidence indicates that DHA supplementation regulates inflammation partially by improving endothelial cell function (Brown and Hu, 2001). Additionally, DHA was shown to significantly decrease cytokine-induced adhesion molecule expression (Chen et al., 2003), diminish the adhesion of leukocytes to activated endothelial cells (Mayer et al., 2002), and to inhibit the production of cytokines by endothelial cells (Von Schacky, 2007).

Heme oxygenase (HO) is an inducible enzyme that catalyzes the rate-limiting step in heme degradation, and produces carbon monoxide (CO), free iron, and biliverdin, which is further catabolized into bilirubin by biliverdin reductase (Otterbein and Choi, 2000). Three isoforms of HO have been identified; these are designated HO-1, HO-2, and HO-3 (Wagener et al., 2003). Induction of the HO-1 protein has been reported to protect against a variety of stressors, such as hydrogen peroxide (Lin et al., 2007), cisplatin (Kim et al., 2006), UV irradiation (Ewing et al., 2005), and inflammatory cytokine-mediated cell damage (Lin et al., 2005). HO-1 was reported to play a part in the pathogenesis and progression of cancers (Jozkowicz et al., 2007). Moreover, HO-1 has been shown to inhibit proliferation and induce apoptosis of several cancers cells, such as breast carcinoma cells (Hill et al., 2005). Furthermore, HO-1 expression is primarily regulated at the transcriptional level, and its induction is linked to the transcriptional factor nuclear factor erythroid 2-related factor 2 (Alam and Cook, 2003). Because of the potent inhibitory role of HO-1 in cancer metastasis and the potential of DHA to induce HO-1 expression (Lu et al., 2010), the anti-tumor properties of DHA have attracted increased research interest in recent years.

Accumulating evidence suggests that bilirubin, free iron, and CO contribute to the protective effects of HO-1 (Gozzelino et al., 2010). HOs are the main producers of CO in the human body. Recently, much attention has been paid to the anti-inflammatory functions of CO (Dulak and Józkowicz, 2003). In addition, CO has been demonstrated to exhibit anti-oxidant, anti-inflammatory, and anti-tumor properties (Nizamutdinova et al., 2009; Chi et al., 2014).

In the present study, we reported that DHA suppressed uPAR expression, and this effect was mediated by HO-1 in human endothelial ECV304 cells. Additionally, CO released from HO-1 catalyzes heme degradation, thereby contributing to HO-1-related inhibition of 12-*O*-tetradecanoylphorbol-13-acetate (TPA)-induced uPAR.

## Materials and Methods

### Reagents

OPTI-modified Eagle’s medium was obtained from HyClone (Logan, UT, United States). TrypLE^™^ Express was obtained from Gibco (Grand Island, NY, United States). TPA, DMSO, tricarbonyldichlororuthenium(II) dimer (RuCO), ruthenium (III) chloride (RuCl_3_), bilirubin, and all other chemicals were purchased from Sigma-Aldrich (St. Louis, MO, United States). Ferric protoporphyrin IX (FePP) and tin protoporphyrin IX (SnPP) were purchased from Santa Cruz Biotechnology (Santa Cruz, CA, United States). The bicinchoninic acid (BCA) protein assay kit was obtained from Pierce (Rockford, IL, United States). Antibodies against uPAR, HO-1, and β-actin were purchased from Cell Signaling Technology (Danvers, MA, United States).

### Cell Culture

The human endothelial cell line ECV304 was cultured in Dulbecco’s modified Eagle’s medium supplemented with 10% fetal bovine serum and 0.6% penicillin-streptomycin (HyClone, Logan, UT, United States). For all experiments, stimulants, such as TPA were added to serum-free media for the indicated time intervals. When inhibitors were used, they were added 1 h before TPA treatment.

### Cell Viability Assay

Cell viability was determined using the MTT assay (Yuan et al., 2018).

### Transient Transfection With siRNAs

Stealth RNAi duplexes corresponding to human siRNAs against HO-1 were purchased from Santa Cruz Biotechnology (Santa Cruz, CA, United States) and transfected into cells with Lipofectamine 2000 reagent (Invitrogen, Carlsbad, CA, United States).

### Luciferase Activity Assay

Cells were seeded and grown until they reached 70% confluency and then co-transfected with siRNAs against HO-1 and PGL3/uPAR. Luciferase activity was performed with Dual-Luciferase Reporter Assay System (Promega, Madison, WI, United States) (Wood et al., 1984; de Wet et al., 1985) according to the manufacturer’s instructions. The plasmid pGL3/uPAR-promoter was supported by Dr. Y. Wang (Australian National University, Canberra, Australia).

### Real-Time Quantitative PCR

Total RNA was isolated from cells using TRIzol reagent (Invitrogen, Carlsbad, CA, United States), and cDNA was synthesized using random primers and M-MLV transcriptase (Promega, Madison, WI, United States) and analyzed by real-time quantitative PCR by SYBR (Applied Biosystems). The PCR conditions and primers for GAPDH and uPAR were reported previously (Lian et al., 2016).

### Western Blotting

Cell lysates were prepared, and Western blot analysis was performed as previously described (Lian et al., 2015).

### Matrigel Invasion Assay

Cell invasion assay was carried out using 10-well chemotaxis chambers (Neuro Probe, Gaithersburg, Maryland, United States) containing an 8 μM pore membrane (Neuro Probe). These chambers contained Dulbecco’s modified Eagle’s medium supplemented with 10% FBS as the chemoattractant in the lower chamber. The non-invading cells on the upper surface of each membrane were removed from the chamber, and the invading cells on the lower surface of each membrane were stained using the Quick-Diff stain kit (Becton-Dickinson, Franklin Lakes, NJ, United States). The number of invading cells was counted using a phase-contrast microscope.

### Statistical Analysis

The column data are shown as the mean ± standard deviation (SD) of at least three experiments. Differences between two data sets were calculated by *t*-tests, and differences among more than two data sets were determined by one-way analysis of variance (ANOVA) using SPSS 17.0 software (IBM, United States). *p* < 0.05 was considered significant.

## Results

### Docosahexaenoic Acid Inhibits 12-*O*-Tetradecanoylphorbol-13-Acetate-Induced Urokinase-Type Plasminogen Activator Receptor Expression in ECV304 Cells

**FIGURE 1:**
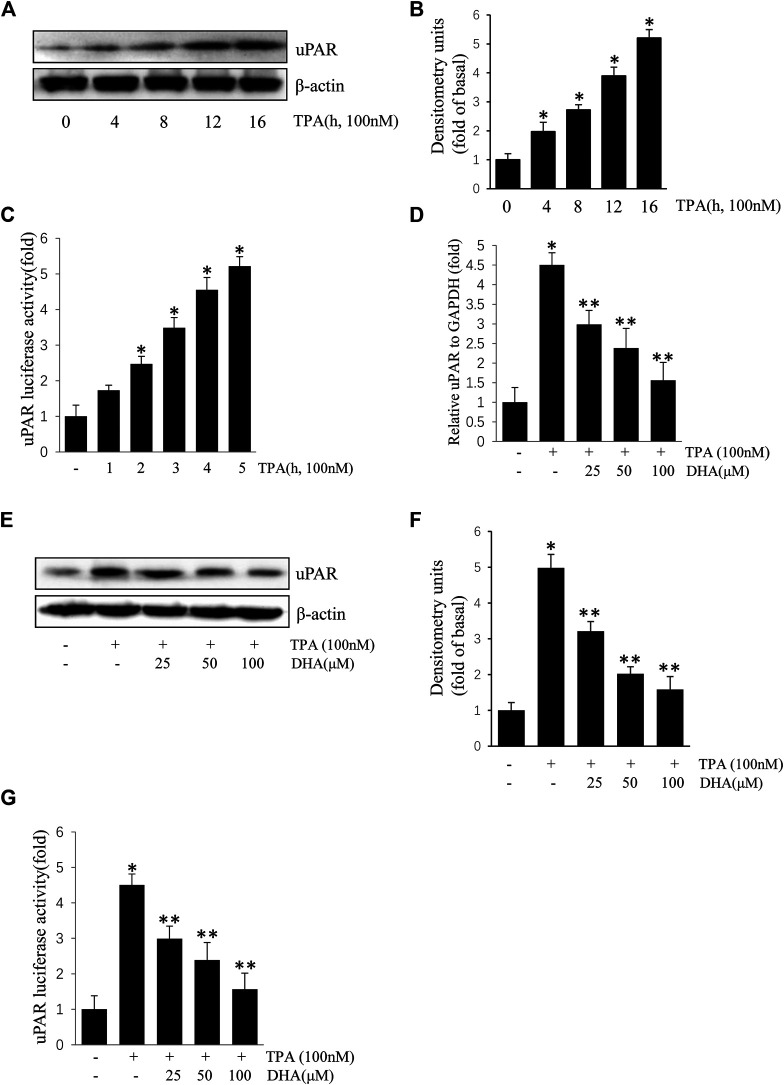
Docosahexaenoic acid suppresses (12-*O*-tetradecanoylphorbol-13-acetate) TPA-induced Urokinase-type plasminogen activator receptor (uPAR) expression in ECV304 cells. **(A–C)** TPA induced the expression of uPAR in a time-dependent manner in ECV304 cells. Cells were treated with 100 nM TPA for 0–16 h. The uPAR protein level **(A,B)** and promoter activity **(C)** were measured by western blotting and luciferase activity assay, respectively. **(D–G)** Cells were pretreated with Docosahexaenoic acid (25, 50, and 100 μM) for 1 h, followed by incubation with 100 nM TPA for 4 or 16 h. The uPAR mRNA level **(D)**, protein level **(E,F)**, and promoter activity **(G)** were measured by real-time PCR, western blotting, and luciferase activity assay, respectively. *p < 0.05 versus control; **p < 0.05 versus TPA treatment alone. Data represent the mean ± SD of triplicate measurements.

ECV304 cells were exposed to 100 nM TPA for 0–16 h. As shown in Figures 1A–C, treatment with TPA increased uPAR protein and mRNA expression in a time-dependent manner. To determine the role of DHA on the increase of uPAR, ECV304 cells pretreated with DHA were incubated with TPA. Results showed that TPA-induced uPAR mRNA expression (Figures 1D), protein expression (Figures 1E,F), and promoter activity (Figures 1G) were inhibited upon pretreatment with by DHA in a dose-dependent manner. DHA did not significantly affect ECV304 cell viability (data not shown). These results suggest that DHA inhibits TPA-induced uPAR expression in ECV304 cells at non-toxic concentrations.

### Docosahexaenoic Acid Inhibits 12-*O*-Tetradecanoylphorbol-13-Acetate-Induced Urokinase-Type Plasminogen Activator Receptor Expression by Inducing Heme Oxygenase-1 Activity

**FIGURE 2:**
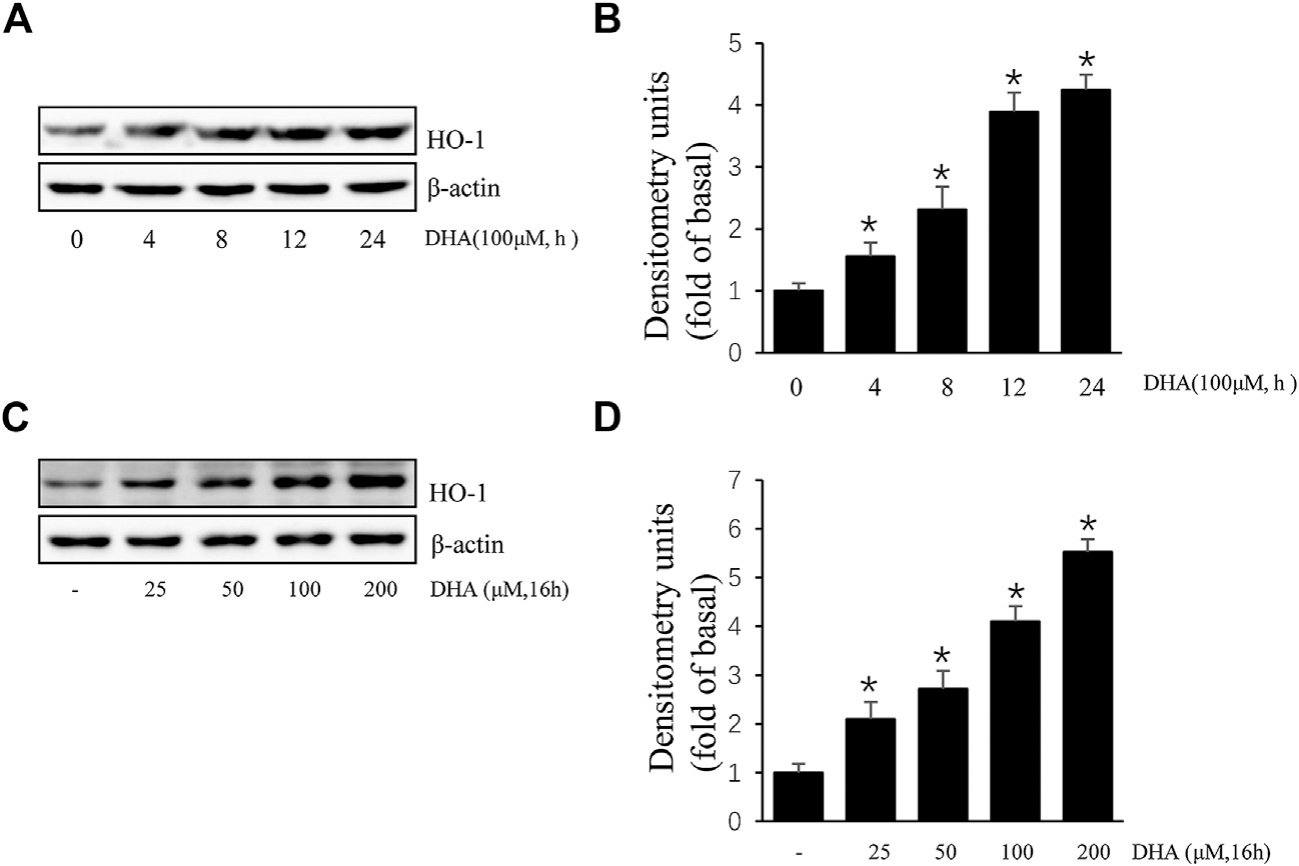
Docosahexaenoic acid (DHA) induces HO-1 expression in ECV304 cells. **(A,B)** DHA induced HO-1 expression in a time-dependent manner. ECV304 cells were treated with 100 μM DHA for 0–24 h, and the cellular extracts were blotted with an antibody against HO-1. **(C,D)** DHA induced HO-1 expression in a dose-dependent manner. ECV304 cells were treated with 0–200 μM DHA for 16 h, and the cellular extracts were blotted using an antibody against HO-1. *p < 0.05 versus control; Data represent the mean ± SD of triplicate measurements.

**FIGURE 3:**
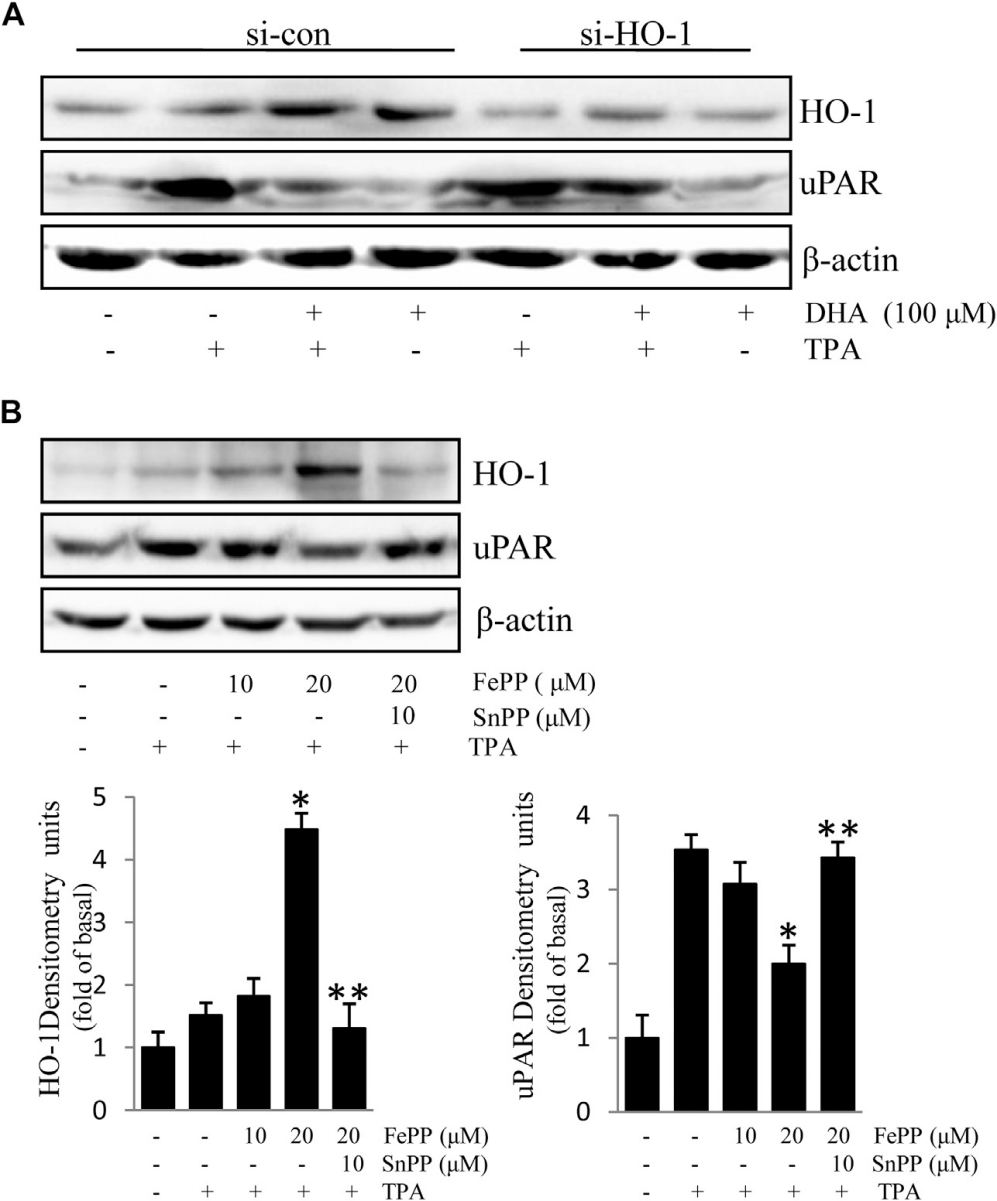
HO-1 is involved in Docosahexaenoic acid-mediated inhibition of (12-*O*-tetradecanoylphorbol-13-acetate) TPA-induced Urokinase-type plasminogen activator receptor (uPAR) expression in ECV304 cells. **(A)** HO-1 siRNA (si-HO-1) transfection was used to silence HO-1 mRNA expression and establish an HO-1 knockdown model in ECV304 cells. Cells were transfected with scrambled (si-con) or si-HO-1 for 24 h, and then treated with 100 μM Docosahexaenoic acid for 4 h before exposure to 100 nM TPA for an additional 16 h. The expression of HO-1 and uPAR was analyzed by western blotting. **(B)** Cells pretreated with FePP (10 and 20 μM) or SnPP (10 μM) for 6 h were incubated with 100 nM TPA for 16 h. The expression of HO-1 and uPAR was analyzed by western blotting. *p < 0.05 versus TPA treatment alone; **p < 0.05 versus FePP treatment. Data represent the mean ± SD of triplicate measurements.

Due to the inhibitory effect of HO-1 on tumor metastasis, agents that can induce HO-1 expression may serve as potential chemotherapeutic drug candidates. To determine whether HO-1 plays a role in DHA-mediated inhibition of TPA-induced uPAR expression, we investigated the effect of DHA on HO-1 expression. As shown in Figure 2, DHA treatment time- and dose-dependently increased HO-1 expression. Furthermore, HO-1 siRNA was used to clarify whether HO-1 is involved in mediating the effects of DHA. Transfection of ECV304 cells with HO-1 siRNA alleviated DHA-induced inhibition of uPAR expression in the presence of TPA (Figures 3A). Next, we examined the effect of the chemical HO-1 inducer FePP on TPA-induced uPAR expression. Interestingly, our results showed an increase in HO-1 protein expression corresponding with a decrease in uPAR expression. Moreover, in the presence of the HO-1 inhibitor SnPP, the inhibition of TPA-induced uPAR expression by FePP was attenuated (Figures 3B). These data suggest that HO-1 is at least partially involved in DHA-mediated inhibition of TPA-induced uPAR expression.

### CO released from HO-1-catalyzed heme degradation is involved in DHA-mediated inhibition of TPA-induced uPAR expression

**FIGURE 4:**
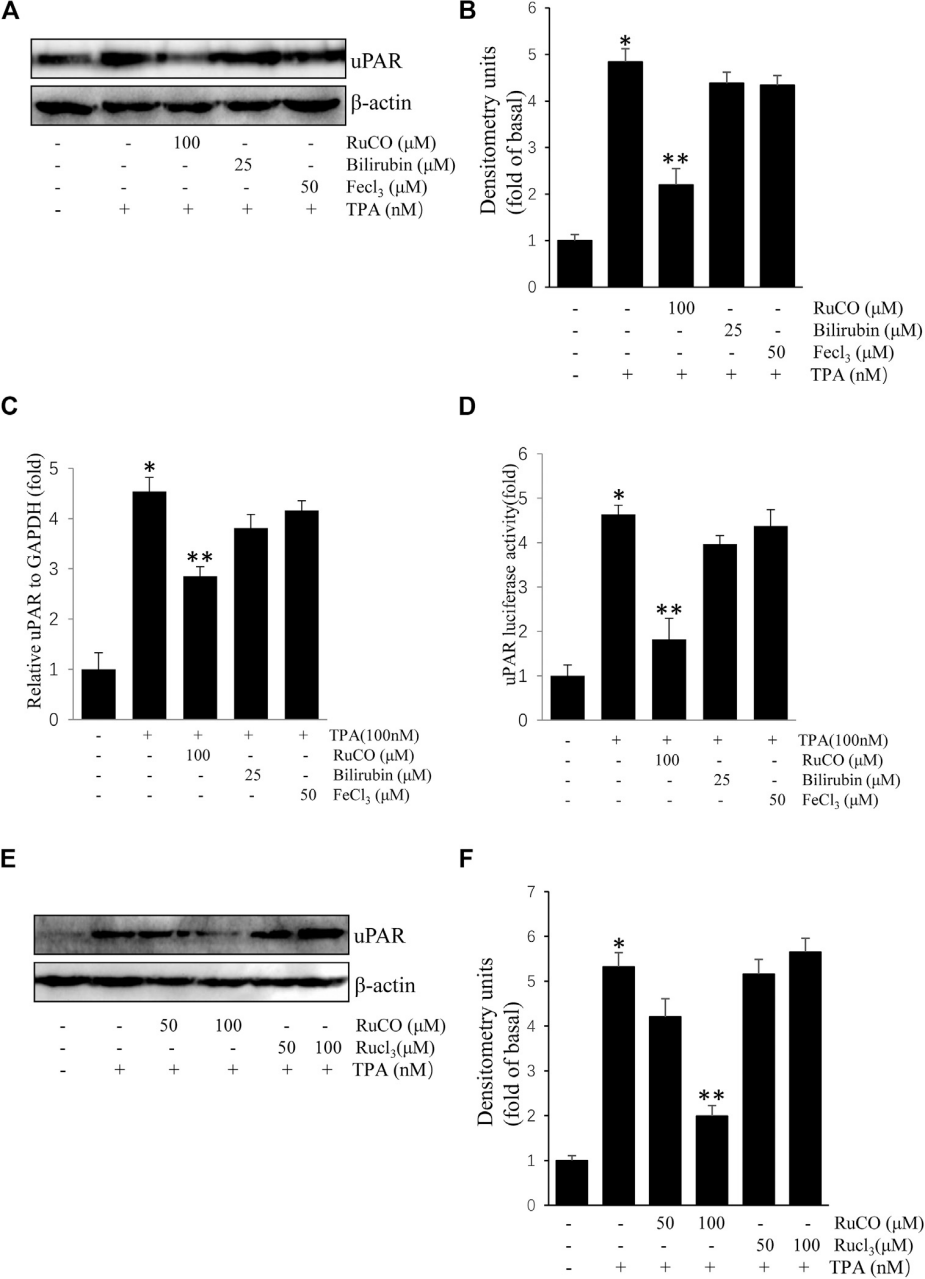
CO inhibits (12-*O*-tetradecanoylphorbol-13-acetate) TPA-induced Urokinase-type plasminogen activator receptor (uPAR) expression. **(A,B)** Cells pretreated with RuCO, bilirubin, or FeCl_3_ for 1 h were incubated with 100 nM TPA for 16 h. The expression of uPAR was analyzed by western blotting. **(C)** Cells pretreated with RuCO or RuCl_3_ for 1 h were incubated with 100 nM TPA for 4 h. The expression of uPAR was analyzed by real-time PCR. **(D)** Cells were transiently transfected with PGL3-uPAR and then treated with RuCO, bilirubin, or FeCl_3_ in the presence of TPA for 6 h, followed by measurement of reporter activity. **(E,F)** Cells pretreated with RuCO or RuCl_3_ for 1 h were incubated with 100 nM TPA for 16 h. The expression of uPAR was analyzed by western blotting. *p < 0.05 versus control; **p < 0.05 versus TPA treatment alone. Data represent the mean ± SD of triplicate measurements.

We used RuCO, FeCl_3_, and bilirubin to determine the individual roles of HO-1 byproducts in TPA-induced uPAR expression. As shown in Figures 4A,B, treatment with FeCl_3_ or bilirubin had no obvious effects on TPA-induced uPAR protein expression. And treatment with FeCl_3_ or bilirubin had also no obvious effects on TPA-induced uPAR mRNA expression (Figures 4C). Next, the effects of HO-1 byproducts on uPAR transcriptional activity were examined using luciferase assay. Results showed that only RuCO pretreatment consistently blocked TPA-induced uPAR promoter activity (Figures 4D). Importantly, the addition of RuCO, but not RuCl_3_, an analogue of RuCO lacking the CO-releasing effect, inhibited uPAR expression (Figures 4E,F). Together, these results imply that CO, but not bilirubin or FeCl_3_ contributes to HO-1-mediated inhibition of TPA-induced uPAR expression.

### Heme Oxygenase-1 is Involved in Docosahexaenoic Acid-Mediated Inhibition of 12-*O*-Tetradecanoylphorbol-13-Acetate-Induced Cell Invasion

**FIGURE 5:**
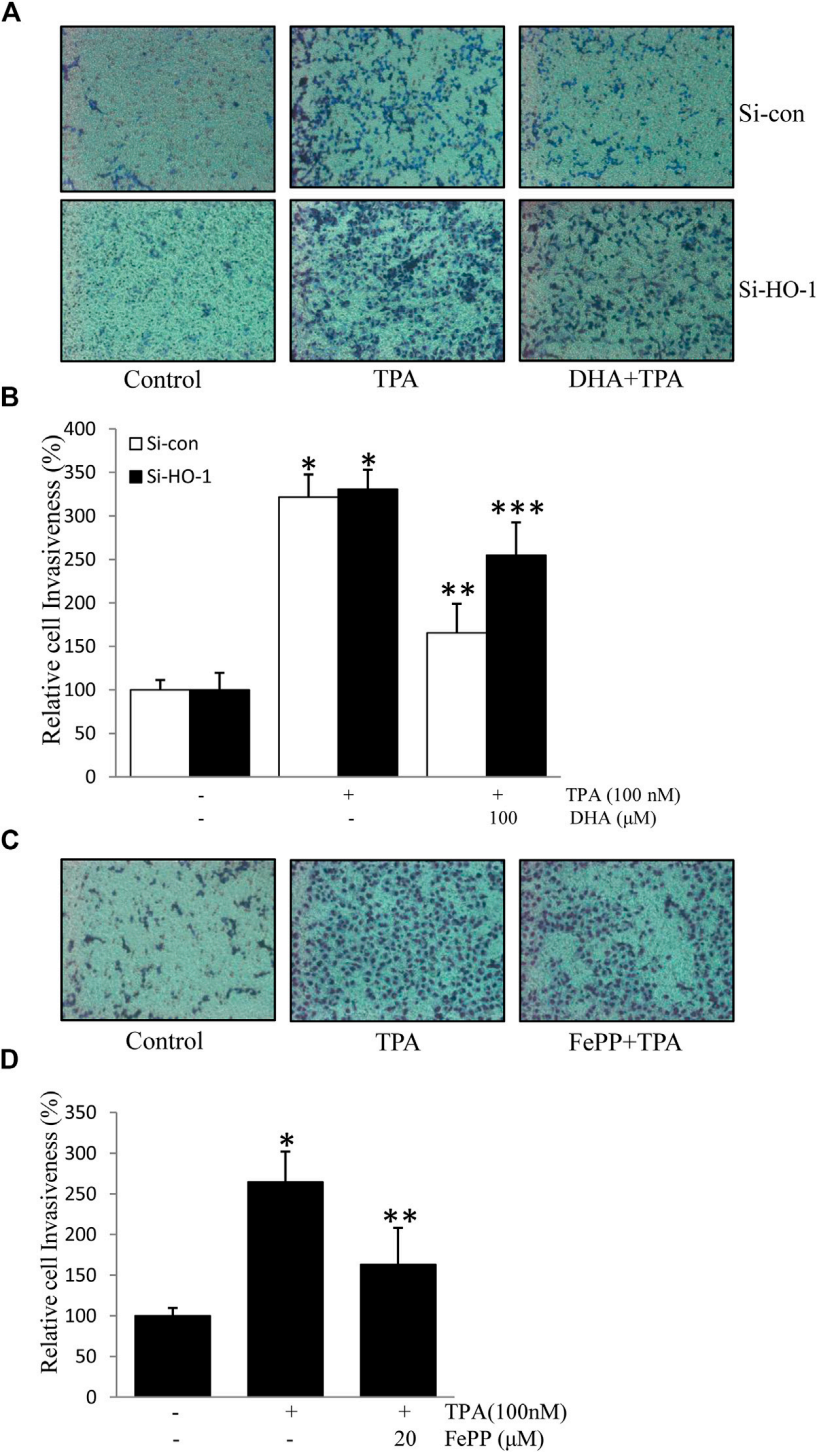
Role of HO-1 on the inhibition of (12-*O*-tetradecanoylphorbol-13-acetate) TPA-induced cell invasion. **(A,B)** Cells (10^5^) transfected with si-HO-1 or si-con were incubated with 100 nM TPA in the presence or absence of 100 μM Docosahexaenoic acid in a BIOCOAT^™^ Matrigel apparatus for 48 h **(C,D)** Cells (10^5^) were incubated with 100 nM TPA in the presence or absence of 20 μM FePP in a BIOCOAT^™^ Matrigel apparatus for 36 h. After incubation, the cells that invaded the undersurface of the chambers were counted using a phase contrast light microscope after staining with Diff-Quick stain. *p < 0.05 versus control; **p < 0.05 versus TPA treatment alone in the si-con group; ***p < 0.05 versus TPA and Docosahexaenoic acid treatments in the si-con group. Data represent the mean ± SD of triplicate measurements.

HO-1, a stress-response protein, has been shown to suppress tumor metastasis (Lin et al., 2008). Cell invasion is a crucial mechanism of tumor metastasis. Here, we first verified the role of HO-1 in cell invasion using HO-1 siRNA. As shown in Figures 5A,B, transfection with si-HO-1 resulted in silencing of HO-1 expression and partially reversed DHA-mediated inhibition of TPA-induced cell invasion, as compared to transfection with control siRNA. Furthermore, we examined the effect of the HO-1 inducer, FePP, on TPA-induced invasion of ECV304 cells. As illustrated in Figures 5A,C,D significant decrease in the number of invasive cells stimulated by TPA was detected in the presence of FePP. These results indicate that HO-1 plays a role in DHA-mediated inhibition of TPA-induced cell invasion.

### Carbon Monoxide Contributes to Docosahexaenoic Acid-Mediated Inhibition of 12-*O*-Tetradecanoylphorbol-13-Acetate-Induced Cell Invasion

**FIGURE 6:**
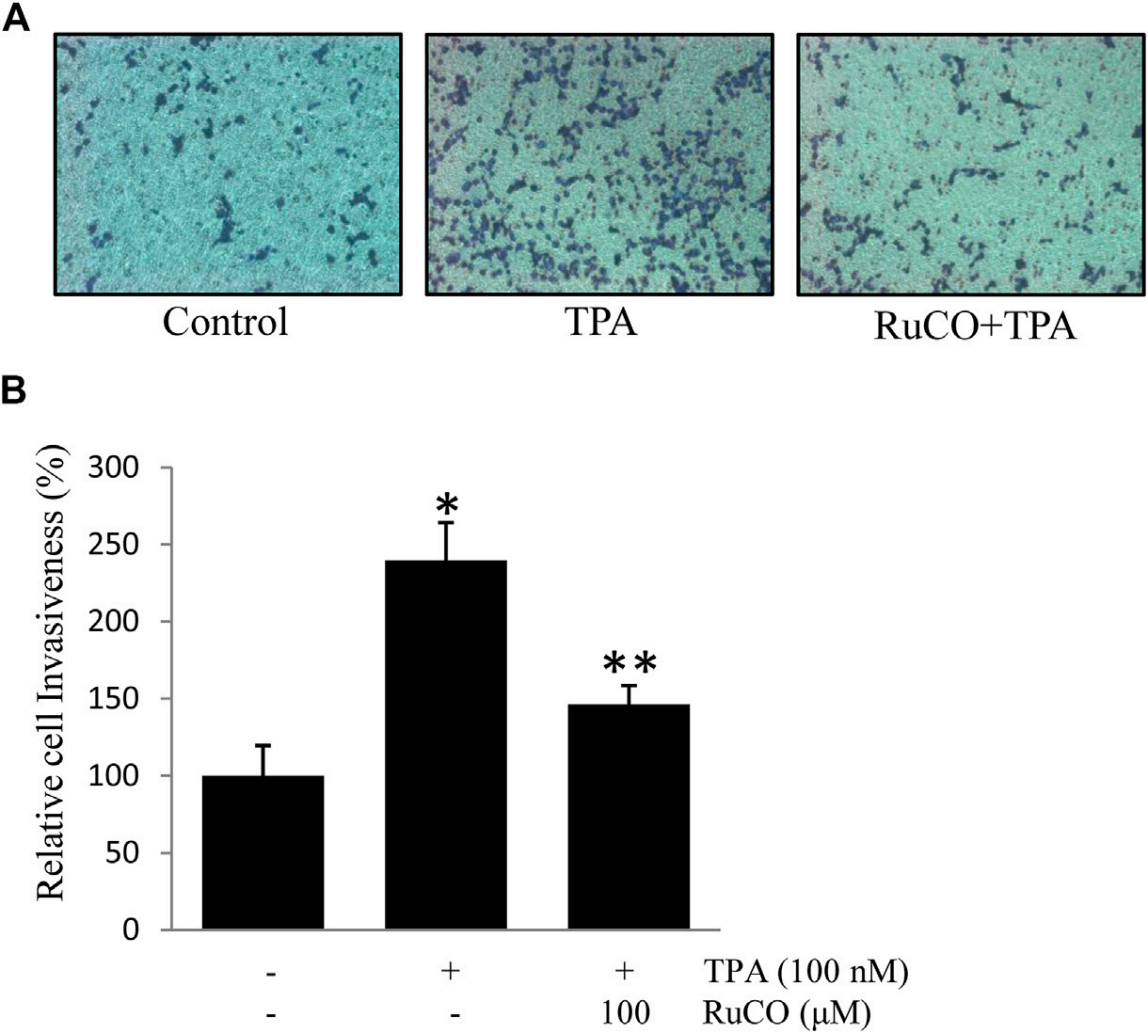
CO contributes to Docosahexaenoic acid-mediated inhibition of (12-*O*-tetradecanoylphorbol-13-acetate) TPA-induced cell invasion. **(A,B)** Cells (10^5^) were incubated with 100 nM TPA in the presence or absence of 100 μM RuCO in a BIOCOAT^™^ Matrigel apparatus for 36 h. Then, the cells that invaded the undersurface of the chambers were counted using a phase contrast light microscope after staining with Diff-Quick stain. *p < 0.05 versus control; **p < 0.05 versus TPA treatment alone. Data represent the mean ± SD of triplicate measurements.

CO is one of the main metabolites of heme degradation by HO-1 and its anti-inflammatory, anti-apoptotic, cytoprotective, and vasodilatory properties have been well documented in different models (Ryter et al., 2006). Accordingly, RuCO (a chemical CO donor) was used to determine the individual role of CO in TPA-induced cell invasion. Interestingly, pretreatment with RuCO significantly inhibited TPA-induced cell invasion (Figures 6A,B). These data suggest that CO released from heme in involved in regulating ECV304 cell invasion.

## Discussion

In recent decades, cancer has attracted considerable attention as it is the leading cause of deaths globally (Garcia et al., 2007). Many studies have been done at defining the role of DHA as a cancer chemopreventive agent in humans, due to various reasons. Firstly, DHA has a preventive and therapeutic effect on cancer (Merendino et al., 2013). In population studies, high intake of fish during many years is associated with decreased risks of colorectal cancer (Reddy, 2004; Cockbain et al., 2012). In breast cancer, DHA has been shown to improve the outcome of chemotheraphy (Menendez et al., 2005). Moreover, ω-3 PUFAs are important constituents of the cell membrane that play multiple roles in regulating membrane fluidity, eicosanoid synthesis, cell signaling, and gene expression (Jump, 2002). DHA is essential for normal brain growth and cognitive function (Nguyen et al., 2014). Accumulating evidence indicates that DHA inhibits the expression of various genes, including VEGF, MMP-9, and COX-2, which are related to inflammation and tumor metastasis (Calviello et al., 2004; Chen et al., 2013; Song et al., 2014). In a previous study, DHA was shown to increase butyrate-mediated apoptosis through promoter methylation (Cho et al., 2014). In this study, we explored the effects of DHA on uPAR expression and cell invasion in human ECV304 endothelial cells. Our results suggested that DHA effectively inhibits TPA-induced uPAR expression and cell invasion, and this effect is likely associated with the upregulation of HO-1.

Induction of antioxidant enzymes is associated with various health benefits. HO-1 expression is considered to be an adaptive and beneficial response to oxidative stress (Takahashi et al., 2004). A previous study indicated that HO-1 induction may increase survival in patients with colorectal cancer by lowering the risk of lymph node metastasis (Becker et al., 2007). In addition, downregulation of HO-1 has been associated with increased malignant progression of hepatocellular carcinoma (Caballero et al., 2004). Recent studies have revealed that HO-1 induction in animals protects against the development of arthritis (Devesa et al., 2005). HO-1 induction was also shown to attenuate the expression of inflammatory cytokines and COX-2 (Kobayashi et al., 2006). Moreover, induction of HO-1 by the chemopreventive agent sulforaphane was demonstrated to contribute to tumor growth suppression by increasing antioxidant response gene expression in hepatoma and breast cancer cells (Keum et al., 2006; Cornblatt et al., 2007). In the current study, treatment with DHA induced HO-1 expression in a time- and dose-dependent manner. However, knockdown of HO-1 by siRNA reversed the DHA-mediated inhibition of TPA-induced uPAR expression. Furthermore, induction of HO-1 by FePP significantly suppressed TPA-induced uPAR expression and cell invasion; however, the suppression of uPAR was attenuated upon treatment with the HO-1 inhibitor, SnPP. These results indicate the importance of HO-1 in DHA-mediated inhibition of TPA-induced uPAR expression. Reactive oxygen species are known to play a crucial role in the etiology of a wide array of human diseases (Kumar et al., 2008). Cells and tissues are routinely subjected to sublethal doses of various oxidants, either exogenously through environmental exposure or endogenously through inflammatory processes (Gopalakrishna and Jaken, 2000; Lagente et al., 2008). HO-1 has been reported to possess anti-oxidant capacity, and HO-1 overexpression was shown to reduce hydrogen peroxide-induced Reactive oxygen species production in macrophages (Lin et al., 2007). In contrast, induction of HO-1 by osteopontin was shown to enhance the migration and invasion of glioma cells (Lu et al., 2012). Kang *et al.* found that HO-1 enhances the resistance of colorectal cancer cells to 5-fluorouracil chemotherapy (Kang et al., 2014). Moreover, in tumor-bearing mice, overexpression of HO-1 was shown to augment melanoma cell viability, proliferation, and angiogenic potential and increase metastasis (Was et al., 2006). Therefore, the role of HO-1 in tumor progression remains controversial. Further *in vitro* and *in vivo* studies are needed to elucidate the role of HO-1 in tumor progression, especially in different types of tumors.

HO-1-catalyzed conversion of heme to CO, free iron, and biliverdin—which is reduced to bilirubin by biliverdin reductase—and the beneficial biological functions of these end products, such as anti-inflammatory, anti-apoptotic, and anti-oxidative effects have been previously reported (Chung et al., 2008; Parfenova et al., 2012). In this study, we used RuCO, FeCl_3_, and bilirubin to verify the role of HO-1 byproducts in TPA-induced uPAR expression and cell invasion. Our results showed that treatment with RuCO, but not bilirubin or FeCl_3_ dose-dependently inhibited TPA-induced uPAR expression. Furthermore, RuCO but not RuCl_3_ possessed the ability to inhibit TPA-induced uPAR expression, suggesting that the metal component Ru is not involved in RuCO-mediated inhibition of uPAR expression. Hence, these findings provide evidence that CO is involved in HO-1-mediated inhibition of TPA-induced cell invasion via suppression of uPAR expression. However, Chao *et al.* had previously reported that not only CO, but also bilirubin and FeCl_3_ inhibit TPA-induced MMP-9 expression in MCF-7 breast cancer cells (Chao et al., 2013).

CO is conventionally recognized as a toxic gas because its binding capacity for heme is four hundred times greater than that for oxygen. Hence, CO poisoning eventually leads to respiratory failure (Foresti et al., 2008). However, CO has been shown to exhibit anti-oxidative, anti-inflammatory and anti-tumor properties. CO induces anti-oxidative effects via inhibition of NADPH oxidase (Taillé et al., 2005). Moreover, CO has also been reported to attenuate inflammatory responses elicited by lipopolysaccharides (Sawle et al., 2005) or cytokines (Megias et al., 2007). Depending on the cell type, CO can activate one or multiple key signaling pathways under various physiological and pathophysiological conditions (Ryter et al., 2006). CO was previously shown to significantly decrease the expression of interleukin-6 by interfering with activator protein-1 activity via the c-Jun N-terminal kinase pathway in lipopolysaccharide-stimulated macrophages (Morse et al., 2003). Moreover, CO has been shown to activate the soluble guanylate cyclase/cyclic guanosine monophosphate pathway, which is implicated in the inhibition of smooth muscle proliferation (Duckers et al., 2001). CO also shows strong potential for use in various therapeutic applications (Ryter et al., 2006). In this respect, the mechanisms underlying CO-mediated inhibition of uPAR expression in ECV304 cells need to be extensively investigated.

In summary, our results suggest that DHA inhibits TPA-induced uPAR expression, and that ECV304 cell invasion is at least partially involved in the induction of HO-1. Moreover, CO released during HO-1-catalyzed heme degradation contributes to the inhibition of TPA-induced uPAR expression. Hence, our data suggest that DHA and HO-1 may serve as potential therapeutic agents or novel target molecules to slow cancer progression.

## Data Availability Statement

The raw data supporting the conclusions of this article will be made available by the authors, without undue reservation.

## Author Contributions

SeL: data curation, investigation, funding acquisition and writing—original draft; ShL and DS: investigation and methodology; NK: resources; VL: conceptualization, resources, supervision, validation, and editing; YJ: conceptualization, funding acquisition, project administration, supervision, validation, writing—review and editing. All authors have read and agreed to the published version of the manuscript.

## Funding

This study was supported by the National Natural Science Foundation of China (no.81702413), the Natural Science Foundation of Guangdong Province, China (2020A1515011433) and Basic Science Research Program grant through the National Research Foundation of Korea funded by the Ministry of Education, Science, and Technology (2018R1D1A1B07049918).

## Conflict of Interest

The authors declare that the research was conducted in the absence of any commercial or financial relationships that could be construed as a potential conflict of interest.
